# The Cardiovascular Risks of Fostamatinib in Patients with Rheumatoid Arthritis: A Systematic Review and Meta-Analysis

**DOI:** 10.3389/fphar.2021.632551

**Published:** 2021-07-19

**Authors:** Yuehong Chen, Huan Liu, Yupeng Huang, Sang Lin, Geng Yin, Qibing Xie

**Affiliations:** Department of Rheumatology and Immunology, West China Hospital, Sichuan University, Chengdu, China

**Keywords:** fostamatinib, rheumatoid arthritis, hypertension, cardiovascular events, meta-analysis, systematic review

## Abstract

**Objective:** This systematic review and meta-analysis is aimed at assessing the risks of cardiovascular adverse events in patients with rheumatoid arthritis (RA) who have been treated with fostamatinib.

**Methods:** The electronic databases of OVID Medline, OVID EMBASE, Cochrane Central Register of Controlled Trials, and Web of Science were searched to identify studies that reported cardiovascular events or hypertension in RA patients treated with fostamatinib. Two reviewers separately and simultaneously screened the retrieved studies based on study selection criteria, collected data and performed methodological quality assessments. The effect size of meta-analysis was estimated by the Peto odds ratio (OR) or relative risk (RR) with 95% confidence intervals (95%CI). Funnel plot was used to estimate publication bias and sensitivity analysis was performed to test the robustness of the results.

**Results:** A total of 12 trials composed of 5,618 participants with low to moderate risk of bias were included. In comparison to the placebo, the use of fostamatinib was found to elevate the risk of hypertension (RR=3.82, 95%CI 2.88–5.05) but was not associated with the risks of all-cause death (Peto OR=0.16, 95%CI 0.02–1.24), major adverse cardiovascular events (Peto OR=1.24, 95%CI 0.26–5.97), pulmonary heart disease and disease of pulmonary circulation (Peto OR=1.23, 95%CI 0.13–11.87), in addition to other forms of heart disease (Peto OR=1.96, 95%CI 0.72–5.38). Furthermore, sensitivity analysis showed no significant change in effective trends and no publication bias was found.

**Conclusion:** Fostamatinib is associated with increased risk of hypertension; however, no increased risks of cardiovascular events were observed. Further well-planned cohort studies with large study populations and longer follow-up times are needed to elucidate the outcomes.

**Systematic Review Registration**: [PROSPERO], identifier [CRD42020198217].

## Introduction

Rheumatoid arthritis (RA) is a chronic systemic autoimmune disease characterized by persistent synovial inflammation and autoantibodies, which mainly occurs among members of the population that are over 40 years of age. Females are more prone to develop RA than males with a ratio of approxiamtely 3:1. The prevalence of RA varies on the basis of country, region, and population. For example, it is 1% for adults in developed countries and 0.2–0.34% for the Chinese (Carmona et al., 2010). RA can influence mental health and cause disability if inappropriately treated, which places a huge economic burden on both families and countries (Fazal et al., 2018; Marrie et al., 2018).

The pathogenesis of RA is largely unknown. It was reported tumor necrosis factor alpha (TNFα), positioning upstream in the cytokine cascade, played a critical role in RA. Correspondingly, anti-TNFα inhibitors are thought to be potent and effective treatments. Nevertheless, nearly half of RA patients who used the anti-TNFα inhibitors failed to reach remission and needed to switch to another anti-TNFα inhibitor or another biologic (Kaltsonoudis et al., 2019). The most effective therapeutic agents anti-TNFα inhibitors for RA patients failed to alleviate disease; therefore, drugs with other targets are needed.

Spleen tyrosine kinase (Syk) plays a critical role in the immune system by mediating inflammatory responses and malignant neoplasms, which makes Syk become a promising drug treatment target for arthritis, asthma, and malignancies like leukemia and lymphoma (Geahlen, 2014). Syk is required for the signaling of macrophages and neutrophils through the Fc gamma (Fcγ) receptor (Kiefer et al., 1998). Macrophages lacking Syk lose the ability to phagocytose IgG-antigen complexes. Notably, after Fcγ receptors recognize IgG-antigen complexes, Syk is activated, which is necessary for the adhesion-dependent activation (Mócsai et al., 2006). Furthermore, the ability of oxidative burst in Syk-deficient neutrophils is lost as oxidative burst in neutrophils is adhesion-dependent. In RA, IgG-self antigen complexes contribute to the pathology by activating macrophages and neutrophils through Rcγ receptors (Kleinau et al., 2000). Therefore, Syk inhibitors can be potential therapeutic drugs for RA.

Fostamatinib is a soluble pro-drug form of R406, a type of Syk inhibitor, and is the most extensively studied Syk inhibitor for RA treatment. It is effective in alleviating the severity of arthritis (Braselmann et al., 2006; Pine et al., 2007) as several clinical trials have proved the effectiveness of fostamatinib in treating RA (Weinblatt et al., 2008; Weinblatt et al., 2010; Genovese et al., 2011). It was reported that RA was associated with an elevated risk of heart failure originating from atherosclerosis (Castaneda et al., 2018; Mackey et al., 2018), while hypertension has been reported to lead to atherosclerosis and cardiovascular disease, resulting in myocardial infarction and stroke (Hollander, 1976). Several published systematic reviews and meta-analyses have reported that fostamatinib can increase the risk of hypertension (Salgado et al., 2014; Kunwar et al., 2016). However, whether or not fostamatinib treatment in RA patients is associated with cardiovascular adverse events is still unknown. Although several systematic reviews and meta-analyses have been performed to synthesize efficacy and safety data for multiple dosages of fostamatinib in RA patients (Salgado et al., 2014; Kunwar et al., 2016; Kang et al., 2019). Nevertheless, none of them focused on the comprehensive evidence of cardiovascular adverse events of fostamatinib. Therefore, this systematic review and meta-analysis was performed to assess the cardiovascular risks of fostamatinib in RA patients as reported by randomized controlled trials (RCTs), cohort studies, or case-control studies.

## Setting

This systematic review and meta-analysis was performed to investigate the cardiovascular risks of fostamatinib in RA patients. The study was registered in PROSPERO (CRD42020198217) and performed on the basis of PRISMA (preferred reporting items for systematic reviews and meta-analyses) guidelines (Moher et al., 2009).

## Methods

### Eligibility Criteria

A study was included if it met all the following requirements: 1): study participants were RA patients; 2); treatment was fostamatinib, regardless of the dose and usage; 3); no limitation for the control treatments, which could be placebo, anti-RA treatments, or fostamatinib with different doses; 4) outcomes were related to cardiovascular events, such as acute myocardial infarction, ischemic stroke, cardiac death, all-cause death, hypertension, or signs related to heart disease; 5); study designs were either RCTs, cohort studies, or case-control studies.

A study was excluded if it was a duplicate, a commentary, a conference abstract, or if it did not have related outcomes.

### Search Strategy

On July 3, 2020, electronic databases of OVID Medline, OVID EMBASE, Web of Science, and Cochrane Central Register of Controlled Trials (CENTRAL) were searched, using both MeSH terms and key words without language limitation. The search terms were “rheumatoid arthritis” and “fostamatinib.” The detailed search strategy can be found in the supplementary document. Reference lists of included studies and the clinicaltrials.gov database were manually checked to identify potentially eligible studies.

### Study Selection

Studies were first screened by titles and abstracts, which were then followed by the reading of full texts based on study selection criteria. Microsoft Office Access 2013 was used to manage study selection. Reference lists and the clinicaltrials.gov database were manually checked. Two reviewers independently and simultaneously screened the studies and any disagreement was resolved *via* discussion or adjudication by a third reviewer if necessary.

### Data Extraction

A pair of two authors independently collected data such as the trial registration number, trial duration, treatments, number of cardiovascular events and cases of hypertension, and number of participants. Any disagreement on data extraction was resolved *via* discussion or adjudication by a third reviewer if necessary.

### Methodological Quality Assessment

Risk of bias of RCTs was assessed using Cochrane Collaboration’s tool for assessing risk of bias (JPT & So, 2011). In doing so, we concentrated on the items of random sequence generation, allocation concealment, blinding of participants and personnel, blinding of outcome assessment, incomplete outcome data, and selective reporting. Each item can by answered by low risk, high risk, or unclear. The risk of bias was judged on the overall evidence. Because no cohort studies or case-control studies were included, the Newcastle–Ottawa scale quality assessment tool was not described here. Methodological quality assessment was simultaneously performed by two authors and any disagreement was resolved *via* discussion or adjudication by a third reviewer if necessary.

### Data Analysis

We performed data analyses using RevMan software (version 5.1.3). The effect size of the meta-analysis was estimated using the relative risk (RR) or Peto Odds Ratio (OR) with 95% confidence intervals (CIs). RR values with 95%CIs were evaluated for the effect size of hypertension using the Mantel-Haenszel fixed effect model, while Peto OR values with 95%CIs were used to assess the effect size of cardiovascular events owing to the low frequency of such events. Clinical diversity across studies, by the statistical heterogeneity, was evaluated using the I-square and heterogeneity *p*-value according to the recommendation by the Cochrane Handbook. I^2^ with values of 25, 50, and 75% indicated low, moderate, and high heterogeneity, respectively (Higgins et al., 2003). Additionally, pre-defined subgroup analyses were conducted by fostamatinib dose and trial duration. Sensitivity analysis using the Mantel-Haenszel random effect model was applied to test the robustness of the results. Furthermore, we assessed the risk of publication bias using funnel plots.

The categorization of cardiovascular diseases was based on the 10th version of the International Classification of Diseases (ICD10)^1^, which includes hypertension (I10–I15), ischemic heart diseases (I20–I25), pulmonary heart disease and disease of pulmonary circulation (I26–I28), other forms of heart disease (I30–I52), and stroke (I64). Major Adverse Cardiovascular Events (MACE) included acute myocardial infarction, ischemic stroke, and cardiac death (Taylor et al., 2019).

## Results

### Study Selection

**FIGURE 1:**
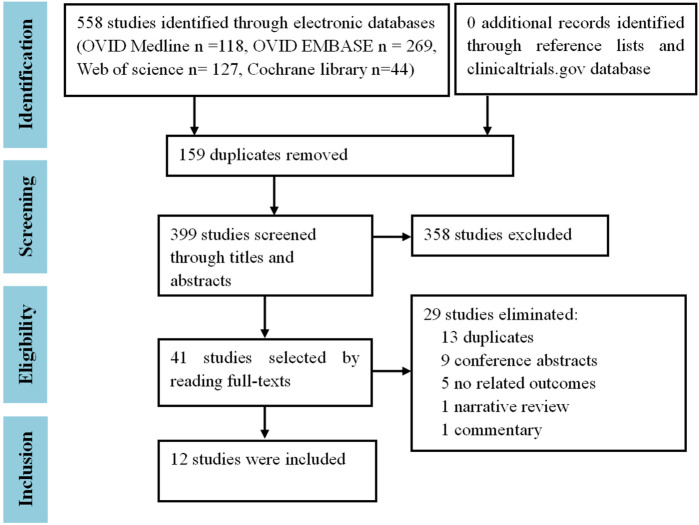
Study selection flow chart.

In total, 558 references were retrieved from the electronic databases of OVID Medline (n = 118), OVID EMBASE (n = 269), Web of Science (n = 127), and the Cochrane library (n = 44). After the removal of 159 duplicates, 399 studies underwent initial screening by titles and abstracts. Thereafter, 358 irrelevant studies were excluded, leaving 41 studies that underwent full-text reading. Finally, 12 trials (^2^ ^,^ ^3^ ^,^ ^4^ ^,^ ^5^ ^,^ ^6^ ^,^ ^7^ ^,^ ^8^ ^,^ ^9^ ^,^ ^10^ ^,^ ^11^ ^,^ ^12^ ^,^ ^13^) were included (Figure 1). No cohort studies or case-control studies were eligible.

### Characteristics of Included Studies

Of the 12 trials, four were phase three studies and eight were phase 2 trials. Trial duration ranged from 4 to 109 weeks. The number of participants varied from 40 to 1,912, and the majority of participants were females, and ages of participants ranged from 50 to 56 years. The geographical study regions were mainly located in the North American, South American, and Europe. Number of study sites ranged from 21 to 310. The common doses and usages of fostamatinib were 100 mg twice daily or 150 mg once daily by oral administration (Table 1).

**TABLE 1:** Characteristics of included RCTs.

Included RCTs	Trial phase	Trial duration	NO. of participants (female/male)	Age (years, mean ± SD)	Study location (number of study sites)	Treatments (participants)
NCT01197521^5^	Phase 3	52 weeks	918 (770/148)	52 ± 12.0	Asia, North America, South America, Europe, Oceanica (127)	Fostamatinib 100 mg twice daily (310)
						Fostamatinib 100 mg twice daily (4 weeks) then 150 mg once daily (304)
						placebo (24 weeks) then fostamatinib 100 mg twice daily (304)
NCT01563978^12^	Phase 2	4 weeks	135 (114/21)	54 ± 12.5	North America, Europe, Africa (59)	Fostamatinib 100 mg twice daily (68)
						placebo (67)
NCT01197534^6^	Phase 3	52 weeks	908 (742/166)	53 ± 11.9	North America, Europe, Asia, Africa (154)	Fostamatinib 100 mg twice daily (308)
						Fostamatinib 100 mg twice daily (4 weeks) then 150 mg once daily (298)
						placebo (24 weeks) then fostamatinib 100 mg twice daily (302)
NCT01197755^7^	Phase 3	24 weeks	322 (261/61)	53 ± 12.3	North America, South America, Europe (136)	Fostamatinib 100 mg twice daily (105)
						Fostamatinib 100 mg twice daily (4 weeks) then 150 mg once daily (108)
						placebo (109)
NCT00665626^3^	Phase 2	12 weeks	219 (176/43)	56.0 ± 11.7	North America, Europe, South America (44)	Fostamatinib 100 mg twice daily (146)
						placebo (73)
NCT01264770^8^	Phase 2b	24 weeks	265 (210/55)	50.0 ± 11.8	North America, Europe, Africa (109)	Fostamatinib 100 mg twice daily (54)
						Fostamatinib 100 mg twice daily (4 weeks) then 150 mg once daily (48)
						Fostamatinib 100 mg twice daily (4 weeks) then 100 mg once daily (57)
						Adalimumab 40 mg every 2 weeks (54)
						placebo (6 weeks) then fostamatinib 100 mg twice daily (27)
						placebo (6 weeks) fostamatinib 100 mg twice daily (4 weeks) then 150 mg once daily (25)
NCT00831922^4^	Phase 2a	12 weeks	40 (9/31)	54.7 ± 10.8	Multicenter, no detailed information	Fostamatinib 3 mg/kg per day (22)
						Fostamatinib 6 mg/kg per day (18)
NCT00326339^13^	Phase 2	12 weeks	189 (164/25)	52.1 (20–75) median	North America (38)	Fostamatinib 50 mg twice daily (46)
						Fostamatinib 100 mg twice daily (49)
						Fostamatinib 150 mg twice daily (47)
						placebo (47)
NCT00665925^2^	Phase 2	26 weeks	457 (390/67)	52.5 ± 12.8	North America, Europe, South America (65)	Fostamatinib 150 mg once daily (152)
						Fostamatinib 100 mg twice daily (152)
						placebo (153)
NCT01242514^9^	Phase 3	109 weeks	1912 (1576/336)	53 ± 11.8	North America, South America, Oceania, Europe, Asia, Africa (310)	Fostamatinib 100 mg twice daily (1343)
						Fostamatinib 100 mg once daily (212)
						Fostamatinib 150 mg once daily (357)
NCT01569074^10^	Phase 2	12 weeks	163 (142/21)	53 ± 11.9	Asia (35)	Fostamatinib 100 mg twice daily (31)
						Fostamatinib 75 mg twice daily (33)
						Fostamatinib 50 mg twice daily (33)
						Fostamatinib 100 mg twice daily (4 weeks) followed by150 mg once daily (33)
						placebo twice daily (33)
NCT02092961^11^	Phase 2	24 weeks	90 (65/25)	51 ± 12.9	North America, europe, Africa (21)	Fostamatinib 100 mg twice daily (33)
						Adalimumab 40 mg SC (28)
						placebo (6 weeks) then fostamatinib 100 mg twice daily (29)

### Methodological Quality

**FIGURE 2:**
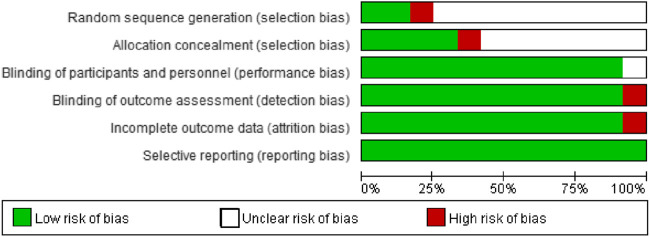
Risk of bias graph.

More than half of the trials did not report detailed methodologies for random sequence generation and allocation concealment. The majority of trials performed the blinding of participants, personnel, and outcome assessment, correctly reported complete outcome data, and all trials did not selectively report outcome data (Figure 2, Supplementary Figure 1). Overall, the risk of bias of included trials was low to moderate.

### Sensitivity Analysis and Publication Bias

**FIGURE 3:**
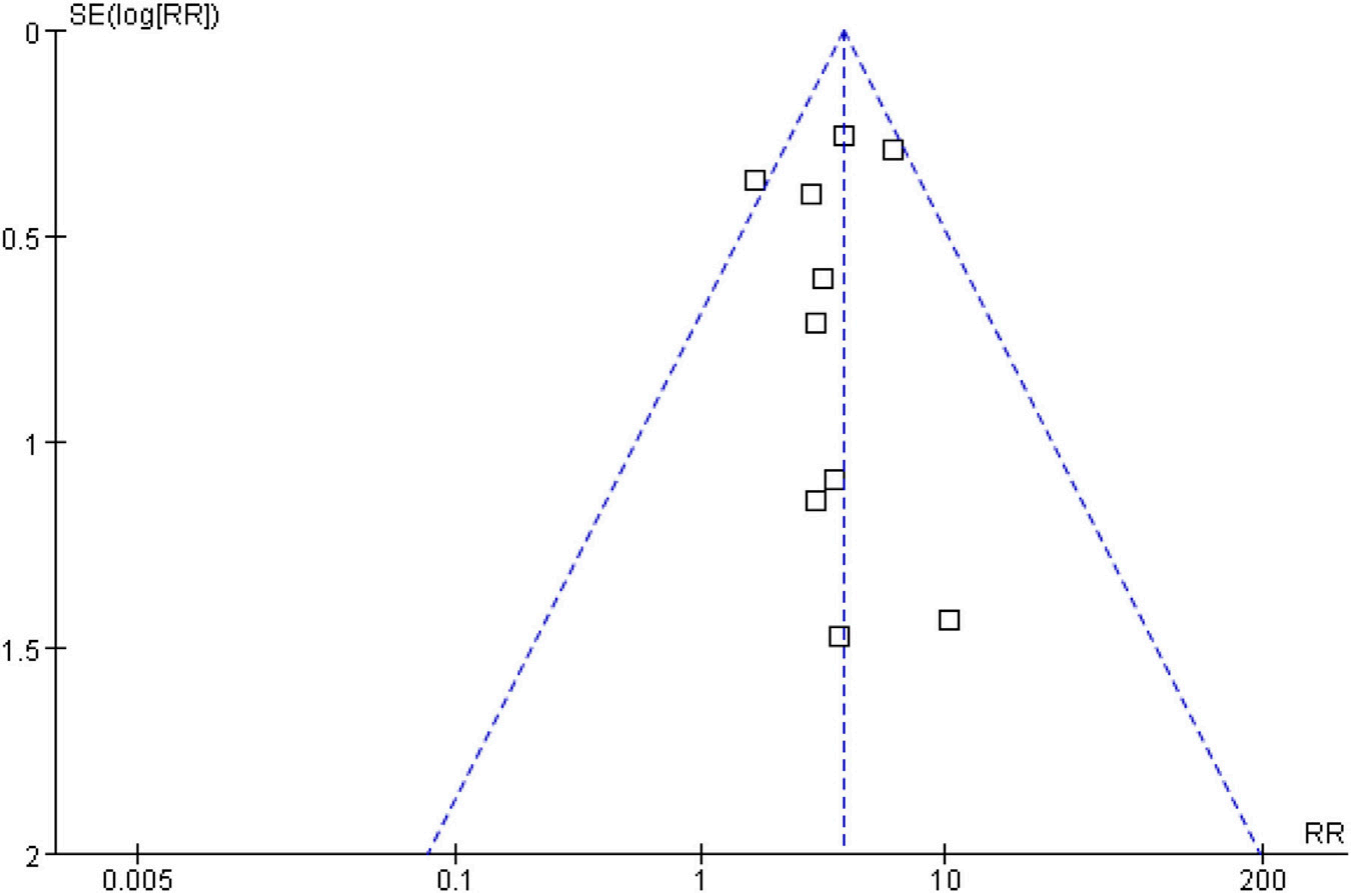
Publication bias based on hypertension.

Sensitivity analysis was performed using the Mantel-Haenszel random effect model to test the robustness of the results and the results showed no important change in effective trend (data not shown). Publication bias, taking the data for hypertension as an example, was assessed by funnel plot. The results showed that the funnel plot was symmetrical, indicating no risk of publication bias (Figure 3).

### Main Outcomes

#### All-Cause Death

One death was reported in 2,437 RA patients treated with fostamatinib. This did not increase all-cause death risk when compared to the placebo group, which had three deaths in 1,187 RA patients (Peto OR = 0.16, 95%CI 0.02–1.24, I^2^ = 0, 1/2437 vs 3/1187) (Table 2, Supplementary Figure 2).

**TABLE 2:** Pooled data of cardiovascular adverse events in RA patients.

Cardiovascular events	Study number	Fostamatinib		Comparator		Heterogeneity		Peto OR, 95% CI
		No. of events	No. of participants	No. of events	No. of participants	I^2^ (%)	*P*	
All-cause death								
Fostamatinib vs PBO	11	1	2,437	3	1,187	0	0.56	0.16 [0.02, 1.24]
MACE								
fostamatinib vs PBO	10	5	2,415	2	1,169	4	0.38	1.24 [0.26, 5.97]
fostamatinib 100 mg bid vs 150 mg qd	9	5	3,007	4	1390	40	0.17	0.48 [0.11, 2.05]
Ischaemic heart disease								
fostamatinib vs PBO	10	6	2,415	2	1,169	0	0.74	1.34 [0.30, 6.03]
fostamatinib 100 mg bid vs 150 mg qd	9	7	3,007	4	1,390	13	0.33	0.81 [0.22, 2.99]
Stroke								
fostamatinib vs PBO	10	2	2,415	1	1,169	33	0.23	1.00 [0.09, 11.02]
fostamatinib 100 mg bid vs 150 mg qd	9	1	3,007	1	1,390	43	0.19	0.31 [0.01, 7.13]
Pulmonary heart disease and disease of pulmonary circulation								
fostamatinib vs PBO	10	2	2,415	0	1,169	0	0.86	1.23 [0.13, 11.87]
fostamatinib 100 mg bid vs 150 mg qd	9	1	3,007	1	1,390	43	0.19	0.31 [0.01, 7.13]
Other forms of heart disease								
fostamatinib vs PBO	10	15	2,415	3	1,169	66	0.02	1.96 [0.72, 5.38]
fostamatinib 100 mg bid vs 150 mg qd	9	13	3,007	8	1,390	67	0.03	0.69 [0.27, 1.76]

#### Major Adverse Cardiovascular Events

MACE includes acute myocardial infarction, ischemic stroke, and cardiac death. When compared to the placebo, fostamatinib did not elevate the MACE risk (Peto OR = 1.24, 95%CI 0.26–5.97, I^2^ = 4, 5/2415 vs 2/1169) (Table 2, Supplementary Figure 3). Moreover, fostamatinib 100 mg administered twice daily did not increase MACE risk than fostamatinib 150 mg administered once daily (Peto OR = 0.48, 95%CI 0.11–2.05, I^2^ = 40, 5/3007 vs 4/1390) (Table 2, Supplementary Figure 4).

### Ischeamic Heart Disease

The use of fostamatinib was not associated with elevated risk of ischemic heart disease, in contrast with the placebo (Peto OR = 1.34, 95%CI 0.30–6.03, I^2^ = 0, 6/2415 vs 2/1169), and fostamatinib 100 mg administered twice daily did not have a higher risk of ischemic heart disease than fostamatinib 150 mg administered once daily (Peto OR = 0.81, 95%CI 0.22–2.99, I^2^ = 13, 7/3007 vs 4/1390) (Table 2, Supplementary Figures 5,6).

#### Stroke

The use of fostamatinib was not correlated with stroke risk (Peto OR = 1.00, 95%CI 0.09–11.02, I^2^ = 33, 2/2415 vs 1/1169) and fostamatinib 100 mg administered twice daily did not relate to increased stroke risk than fostamatinib 150 mg administered once daily (Peto OR = 0.31, 95%CI 0.01–7.13, I^2^ = 43, 1/3007 vs 1/1390) (Table 2, Supplementary Figures 7,8).

### Other Forms of Heart Disease

Fostamatinib did not increase the risks of pulmonary heart disease and disease of pulmonary circulation (Peto OR = 1.23, 95%CI 0.13–11.87, I^2^ = 0, 2/2415 vs 0/1169) (Table 2, Supplementary Figure 9), as well as other forms of heart disease (Peto OR = 1.96, 95%CI 0.72–5.38, I^2^ = 66, 15/2415 vs 3/1169) (Table 2, Supplementary Figure 11), in comparison with the placebo. Specifically, fostamatinib 100 mg administered twice daily did not elevate the risks of pulmonary heart disease and disease of pulmonary circulation (Peto OR = 0.31, 95%CI 0.01–7.13, I^2^ = 43, 1/3007 vs 1/1390) (Table 2, Supplementary Figure 10), as well as other forms of heart disease, compared to fostamatinib 150 mg administered once daily (Peto OR = 0.69, 95%CI 0.27–1.76, I^2^ = 67, 13/3007 vs 8/1390) (Table 2, Supplementary Figure 12).

### Hypertension

The use of fostamatinib elevated the risk of hypertension when compared to the placebo (RR = 3.82, 95%CI 2.88–5.05, I^2^ = 3, 408/2415 vs 51/1169) (Table 3, Supplementary Figure 13). This increased risk was not observed in comparison to adamumab (RR = 1.66, 95%CI 0.71–3.91, I^2^ = 0, 31/266 vs 5/82) (Table 3, Supplementary Figure 16). Fostamatinib 100 mg administered twice daily was associated with increased hypertension risk than 100 mg administered once daily (RR = 1.98, 95%CI 1.26–3.11, I^2^ = 0, 208/1424 vs 20/269) (Table 3, Supplementary Figure 15). Nevertheless, fostamatinib 100 mg administered twice daily did not cause a higher risk of hypertension than 150 mg administered once daily (RR = 0.94, 95%CI 0.80–1.10, I^2^ = 79, 433/3097 vs 205/1372) (Table 3, Supplementary Figure 14).

**TABLE 3:** Pooled data of hypertension in RA patients.

Comparisons	Study number	Fostamatinib		Comparator		Heterogeneity		RR, 95% CI
		No. of events	No. of participants	No. of events	No. of participants	I^2^ (%)	*p*	
Fostamatinib vs PBO	10	408	2,415	51	1,169	3	0.41	3.82 [2.88, 5.05]
4 weeks	1	3	68	1	67	—	—	2.96 [0.32, 27.71]
12 weeks	3	47	418	5	153	0	0.99	3.12 [1.32, 7.38]
24 weeks	4	87	709	17	343	0	0.48	2.48 [1.51, 4.10]
52 weeks	2	271	1,220	28	606	31	0.23	4.81 [3.30, 7.01]
Fostamatinib 100 mg bid vs 150 mg qd	8	433	3,097	205	1,372	79	<0.0001	0.94 [0.80, 1.10]
12 weeks	2	20	192	8	80	0	0.79	0.91 [0.43, 1.95]
24 weeks	3	46	338	43	333	0	0.90	1.05 [0.72, 1.55]
52 weeks	2	170	1,224	131	602	53	0.14	0.64 [0.52, 0.78]
109 weeks	1	197	1,343	23	357	—	—	2.28 [1.50, 3.45]
Fostamatinib 100 mg bid vs 100 mg qd	2	208	1,424	20	269	0	0.61	1.98 [1.26, 3.11]
Fostamatinib vs ADA	2	31	266	5	82	0	0.33	1.66 [0.71, 3.91]

Subgroup analysis revealed that longer periods of fostamatinib use were correlated with hypertension risk. When compared to the placebo, the use of fostamatinib increased hypertension risk at 12 weeks (RR = 3.12, 95%CI 1.32–7.38), 24 weeks (RR = 2.48, 95%CI 1.51–4.10), and 52 weeks (RR = 4.81, 95%CI 3.30–7.01) (Table 3, Supplementary Figure 13). When compared to fostamatinib 150 mg administered once daily, the use of fostamatinib 100 mg administered twice daily had a higher hypertension risk at 109 weeks (RR = 2.28, 95%CI 1.50–3.45) (Table 3, Supplementary Figure 14).

## Discussion

### Main Findings

To our knowledge, this is the first systematic review and meta-analysis to assess the risk of cardiovascular adverse events in RA patients treated with fostamatinib and the results suggest that fostamatinib is associated with increased hypertension risk, when compared to the placebo, and longer periods and higher doses of use are related to higher risk. However, fostamatinib is considered safe for the cardiovascular system as fostamatinib does not increase the risks of all-cause death, MACE, ischemic heart disease, stroke, pulmonary heart disease and disease of pulmonary circulation, in addition to other forms of heart disease.

The indications of fostamatinib are, in addition to RA, conditions such as acute myeloid leukemia, chronic lymphocytic leukemia, lymphoma, immune thrombocytopenia, autoimmune hemolytic anemia, and IgA nephropathy (Friedberg et al., 2010; Morales-Torres, 2010; Efremov & Laurenti, 2011; Bartaula-Brevik et al., 2018; Markham, 2018). Overall, fostamatinib is generally well tolerated, but the most common adverse events include hypertension, diarrhea, dizziness, nausea, epistaxis, abnormal liver function tests, and infections, which are mild or moderate and can be resolved by medication or relieved spontaneously (Bussel et al., 2018; Bussel et al., 2019; Moore et al., 2019).

It was reported that overexpression of the Syk gene could accelerate the development of atherosclerosis (Guo et al., 2019); therefore, the use of fostamatinib to inhibit Syk may retard the development of atherosclerosis. On the contrary, fostamatinib was found to be associated with hypertension, which is a major risk factor for cardiovascular events. Nevertheless, no protective or increased risks were observed in regard to cardiovascular adverse events in our study. This can be explained by the fact that, on the one hand, it may take a longer time from the development of hypertension to atherosclerosis then to cardiovascular events like myocardial infarction and stroke. As such, the follow-up duration of the trials may not be long enough. On the other hand, the sample size may be too small to achieve a statistically significant difference. Therefore, the cardiovascular adverse events of fostamatinib in RA should be of concern.

Several published systematic reviews and meta-analyses also concerned the safety of fostamatinib for RA patients. Nevertheless, none of them focused on cardiovascular adverse events except for hypertension, as it had been reported that fostamatinib was associated with increased risk of hypertension (Kang et al., 2019). Kang et al., 2019 reported that in comparison to the placebo, fostamatinib was found to have increased risks of serious adverse events (all doses: RR = 2.10, 95%CI 1.57–2.80; 100 mg twice daily: RR = 2.10, 95%CI 1.56–2.83) and other adverse events (all doses: RR = 1.63, 95%CI 1.33–2.01; 100 mg twice daily: RR = 1.79, 95%CI 1.44–2.22) (Kang et al., 2019). Kunwar et al., 2016 suggested that fostamatinib was associated with increased risks of infection (OR = 1.59, 95%CI 1.2–2.11), diarrhea (OR = 3.54, 95%CI 2.43–5.16), hypertension (OR = 2.55, 95%CI 1.54–4.22), and neutropenia (OR = 5.68, 95%CI 1.97–16.42) (Kunwar et al., 2016). Similarly, Salgado et al., 2014 suggested that fostamatinib increased the risks of hypertransaminasemia (RR = 2.93, 95%CI 1.02–8.43), hypertension (RR = 2.80, 95%CI 1.58–5.99), diarrhea (RR = 5.20, 95%CI 3.19–8.49), and neutropenia (RR = 9.24, 95%CI 2.22–38.42) (Salgado et al., 2014). Our study reports that fostamatinib is associated with increased hypertension risk, which is related to period of use, with longer periods of use carrying higher risks.

### Limitations

Several limitations of the systematic reviews and meta-analyses performed within this study should be considered. Firstly, several trials did not describe the method with how the random sequence generation was done nor did they detail how allocation concealment was performed. Based on Cochrane Collaboration’s tool for assessing risk of bias, the correct method of random sequence generation involves using a random number table, a computer random number generator, or by coin tossing. Almost all trials described the allocation concealment as random, but the majority of them did not give a detailed description such as whether it occurred *via* the telephone, was web-based, or done using sealed envelopes. Secondly, the eligible study number and sample size are small. Overall, only thousands of participants were enrolled and only one study was eligible to be a part of several subgroup analyses. Moreover, several trials did not have full-text links, which may compromise the study quality when assessing the risk of bias. Finally, only RCTs met the study selection criteria that were included, while no cohort studies or case-control studies were eligible. In total, the small study population and relatively short follow-up duration of the RCTs were not necessarily powered for detecting infrequent adverse events, such as non-hypertension cardiovascular events (e.g., only one event of a stroke from 1,169 patients receiving placebo), nor were there sufficient follow-up durations to detect adverse events arising from hypertension. Therefore, observational studies, such as cohort studies, with long follow-up durations and large study populations are required to rule out possible cardiovascular adverse events and all-cause death of fostamatinib in RA patients.

## Conclusion

This study suggests that the use of fostamatinib increases the risk of hypertension in a time-and dose-dependent way. Nevertheless, fostamatinib is not found to be associated with cardiovascular adverse events such as MACE, ischemic heart disease, and stroke. Well-planned observation studies, such as cohort studies, with large populations and long follow-up periods are needed to elucidate the relationship between fostamatinib and the risk of cardiovascular events owing to the limitations of the present study.

## Data Availability

The original contributions presented in the study are included in the article/Supplementary Material, further inquiries can be directed to the corresponding authors.

## Footnotes

1 WHO:the 10th Revision of the International Classification of Diseases (ICD-10). Available from: http://www.cdc.gov/nchs/icd/icd10cm.htm.

2 Efficacy and Safety Study of R935788 Tablets to Treat Rheumatoid Arthritis (Taski-2). https://www.clinicaltrials.gov/ct2/show/NCT00665925?term=NCT00665925&draw=2&rank=1.

3 Efficacy and Safety Study of R935788 Tablets to Treat Rheumatoid Arthritis (Taski-3). https://www.clinicaltrials.gov/ct2/show/NCT00665626?term=NCT00665626&draw=2&rank=1.

4 Efficacy of Oral AB1010 in Adult Patients With Active Rheumatoid Arthritis. https://www.clinicaltrials.gov/ct2/show/NCT00831922?term=NCT00831922&draw=2&rank=1.

5 Evaluation of Effectiveness of Two Dosing Regimens of fostamatinib Compared to Placebo in Patients With Rheumatoid Arthritis (RA) Who Are Taking Methotrexate But Not Responding (OSKIRA - 1). https://www.clinicaltrials.gov/ct2/show/NCT01197521?term=NCT01197521&draw=2&rank=1.

6 Evaluation of Effectiveness of Two Dosing Regimens of fostamatinib Compared to Placebo in Patients With Rheumatoid Arthritis (RA) Who Are Taking disease Modifying Anti-rheumatic Drug (DMARD) But Not Responding (OSKIRA - 2). https://www.clinicaltrials.gov/ct2/show/NCT01197534?term=NCT01197534&draw=1&rank=1.

7 Evaluation of Effectiveness of Two Dosing Regimens of fostamatinib Compared to Placebo in Patients With Rheumatoid Arthritis (RA) Who Are Taking Methotrexate and Have Had Inadequate Response to Single TNF-alpha Antagonist (OSKIRA - 3). https://www.clinicaltrials.gov/ct2/show/NCT01197755?term=NCT01197755&draw=2&rank=1.

8 Evaluation of Efficacy and Safety of fostamatinib Monotherapy Compared With adalimumab Monotherapy in Patients With Rheumatoid Arthritis (RA) (OSKIRA -4). https://www.clinicaltrials.gov/ct2/show/NCT01264770?term=NCT01264770&draw=1&rank=1.

9 Evaluation of Long-term Safety and Effectiveness of fostamatinib in the Treatment of Rheumatoid Arthritis (RA) (OSKIRA-X). https://www.clinicaltrials.gov/ct2/show/NCT01242514?term=NCT01242514&draw=2&rank=1.

10 Evaluation of Safety and Effectiveness of fostamatinib Compared to Placebo in Patients in Asia With Rheumatoid Arthritis (OSKIRA-Asia-1). https://www.clinicaltrials.gov/ct2/show/NCT01569074?term=NCT01569074&draw=1&rank=1.

11 Randomised Double-Blind, Placebo-Controlled, Parallel Group Study in Patients With Active Rheumatoid Arthritis:Magnetic Resonance Imaging Sub-Study (OSKIRA 4 SS). https://www.clinicaltrials.gov/ct2/show/NCT02092961?term=NCT02092961&draw=2&rank=1.

12 Study of the Effect of fostamatinib Twice Daily on Blood Pressure in Patients With Rheumatoid Arthritis (Oskira ABPM). https://www.clinicaltrials.gov/ct2/show/NCT01563978?term=NCT01563978&draw=2&rank=1.

13 Treatment of Arthritis With Syk kinase Inhibition (TASKI-1). https://www.clinicaltrials.gov/ct2/show/NCT00326339?term=NCT00326339&draw=1&rank=1.
