# Supplementary material for: The Cardiovascular Risks of Fostamatinib in Patients with Rheumatoid Arthritis: A Systematic Review and Meta-Analysis
Source: Front Pharmacol. 2021 Jul 19;12:632551. doi: 10.3389/fphar.2021.632551 (PMC8327174; doi:10.3389/fphar.2021.632551)
Supplement: Supplementary file 1 [file datasheet2.pdf]

Detailed Search Strategy

The cardiovascular risks of fostamatinib in patients with rheumatoid arthritis : a systematic review and meta-analysis

Yuehong Chen, Huan Liu, Yupeng Huang, Sang Lin, Geng Yin*, Qibing Xie#

Department of Rheumatology and Immunology, West China Hospital, Sichuan University, Chengdu 610041, China

* Co-corresponding author, email: yingeng1975@163.com, Department of Rheumatology and Immunology, West China Hospital, Sichuan University, 37 Guoxue lane, Chengdu 610041, China, Tel/Fax: +86-28-8542 2393

# Corresponding author, email: xieqibing1971@163.com, Department of Rheumatology and Immunology, West China Hospital, Sichuan University, 37 Guoxue lane, Chengdu 610041, China, Tel/Fax: +86-28-8542 2393

Search date July 3, 2020
Ovid MEDLINE(R) 1946 to June Week 4 2020 (n=118)
Ovid EMBASE 1974 to 2020 July 02 (n=269)
Web of science n=127
Cochrane libraey n=44

Database: Ovid MEDLINE(R) <1946 to June Week 4 2020>
Search Strategy:
1 rheumatoid arthritis.mp. or exp Arthritis, Rheumatoid/
2 ((rheumatoid or reumatoid or revmatoid or rheumatic or reumatic or revmatic or rheumat$ or reumat$ or revmarthrit$) adj3 (arthrit$ or artrit$ or diseas$ or condition$ or nodule$)).tw.
3 (felty$ adj2 syndrome).tw.
4 (caplan$ adj2 syndrome).tw.
5 (sjogren$ adj2 syndrome).tw.
6 (sicca adj2 syndrome).tw.
7 still$ disease.tw.
8 bechterew$ disease.tw.
9 1 or 2 or 3 or 4 or 5 or 6 or 7 or 8
10 Fostamatinib.mp.
11 Fostamatinib Disodium.mp.
12 Syk Kinase/
13 Tavalisse.mp.
14 Masitinib.mp.
15 AB1010.mp.
16 ab1010.mp.
17 AB 1010.mp.
18 ab 1010.mp.
19 AB-1010.mp.
20 UNII-M59NC4E26P.mp.
21 R-788.mp.
22 r-788.mp.
23 R 788.mp.
24 R788.mp.
25 tamatinib fosdium.mp.
26 R935788.mp.
27 R 935788.mp.
28 R-935788.mp.
29 R-788$400.mp.
30 R 788$400.mp.
31 R788$400.mp.
32 10 or 11 or 12 or 13 or 14 or 15 or 16 or 17 or 18 or 19 or 20 or 21 or 22 or 23 or 24 or 25 or 26 or 27 or 28 or 29 or 30 or 31
~~34~~ 9 and 32
33

Database: Embase <1974 to 2020 July 02>
Search Strategy:
1 rheumatoid arthritis.mp. or exp rheumatoid arthritis/
2 ((rheumatoid or reumatoid or revmatoid or rheumatic or reumatic or revmatic or rheumat$ or reumat$ or revmarthrit$) adj3 (arthrit$ or artrit$ or diseas$ or condition$ or nodule$)).tw.
3 (felty$ adj2 syndrome).tw.
4 (caplan$ adj2 syndrome).tw.
5 (sjogren$ adj2 syndrome).tw.
6 (sicca adj2 syndrome).tw.
7 still$ disease.tw.
8 bechterew$ disease.tw.
9 1 or 2 or 3 or 4 or 5 or 6 or 7 or 8
10 Fostamatinib.mp. or exp fostamatinib/
11 Fostamatinib Disodium.mp.
12 Tavalisse.mp.
13 Masitinib.mp.
14 AB1010.mp.
15 ab1010.mp.
16 AB 1010.mp.
17 ab 1010.mp.
18 AB-1010.mp.
19 UNII-M59NC4E26P.mp.
20 R-788.mp.
21 r-788.mp.
22 R 788.mp.
23 R788.mp.
24 tamatinib fosdium.mp.
25 R935788.mp.
26 R 935788.mp.
27 R-935788.mp.
28 R-788-400.mp.
29 R-788$400.mp.
30 R 788$400.mp.
31 R788$400.mp.
32 10 or 11 or 12 or 13 or 14 or 15 or 16 or 17 or 18 or 19 or 20 or 21 or 22 or 23 or 24 or 25 or 26 or 27 or 28 or 29 or 30 or 31
33 9 and 32

Database: Web of science (1900-2020)
1 TS=(rheumatoid arthritis)
2 ALL=((rheumatoid or reumatoid or revmatoid or rheumatic or reumatic or revmatic or rheumat$ or reumat$ or revmarthrit$) adj3 (arthrit$ or artrit$ or diseas$ or condition$ or nodule$)).tw.)
3 1 or 2
4 TS=(Fostamatinib)
5 TS=(Fostamatinib Disodium)
6 ALL=(Fostamatinib)
7 ALL=(Tavalisse)
8 ALL=(Masitinib)
9 ALL=(AB1010)
10 ALL=(AB 1010)
11 ALL=(AB-1010)
12 ALL=(R-788)
13 ALL=(R 788)
14 ALL=(R788)
15 ALL=(tamatinib fosdium)
16 ALL=(Fostamatinib Disodium)
17 ALL=(R935788)
18 ALL=(R 935788)
19 ALL=(R-935788)
20 ALL=(R-788-400)
21 ALL=(R-788$400)
22 ALL=(R 788$400)
23 ALL=(R788$400)
24 ALL=()
25 4 or 5 or 6 or 7 or 8 or 9 or 10 or 11 or 12 or 13 or 14 or 15 or 16 or 17 or 18 or 19 or 20 or 21 or 22 or 23 or 24
26 3 and 25

Cochrane library

1 MeSH descriptor: [Arthritis, Rheumatoid] explode all trees
2 (rheumatoid arthritis):ti,ab,kw
3 (felty$ adj2 syndrome):ti,ab,kw
4 (caplan$ adj2 syndrome):ti,ab,kw
5 (sjogren$ adj2 syndrome):ti,ab,kw
6 (sicca adj2 syndrome):ti,ab,kw
7 still$ disease:ti,ab,kw
8 bechterew$ disease:ti,ab,kw
9 1 or 2 or 3 or 4 or 5 or 6 or 7 or 8
10 (Fostamatinib):ti,ab,kw
11 (Fostamatinib Disodium):ti,ab,kw
12 (Tavalisse):ti,ab,kw
13 (Masitinib):ti,ab,kw
14 (AB1010):ti,ab,kw
15 (ab1010):ti,ab,kw
16 (AB 1010):ti,ab,kw
17 (ab 1010):ti,ab,kw
18 (AB-1010):ti,ab,kw
19 (R-788):ti,ab,kw
20 (r-788):ti,ab,kw
21 (R 788):ti,ab,kw
22 (R788):ti,ab,kw
23 (tamatinib fosdium):ti,ab,kw
24 (R935788):ti,ab,kw
25 (R 935788):ti,ab,kw
26 (R-935788):ti,ab,kw
27 (R-788-400):ti,ab,kw
28 (R-788$400):ti,ab,kw
29 (R 788$400):ti,ab,kw
30 (R788$400):ti,ab,kw
31 10 or 11 or 12 or 13 or 14 or 15 or 16 or 17 or 18 or 19 or 20 or 21 or 22 or 23 or 24 or 25 or 26 or 27 or 28 or 29 or 30
32 9 and 31
