# Supplementary material for: The Cardiovascular Risks of Fostamatinib in Patients with Rheumatoid Arthritis: A Systematic Review and Meta-Analysis
Source: Front Pharmacol. 2021 Jul 19;12:632551. doi: 10.3389/fphar.2021.632551 (PMC8327174; doi:10.3389/fphar.2021.632551)
Supplement: Supplementary file 2 [file datasheet1.pdf]

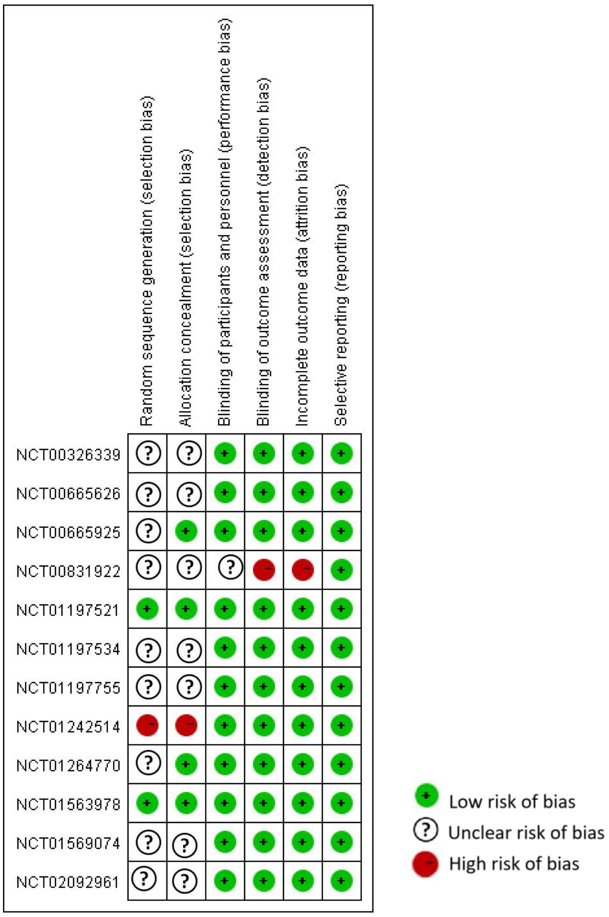

sFig.1 Risk of bias summary: each risk of bias item for each included study.

Study or Subgroup	Fostamatinib Events	Fostamatinib Total	Placebo Events	Placebo Total	Weight	Peto Odds Ratio Peto, Fixed, 95% CI
NCT00326339	0	142	0	47		Not estimable
NCT00665626	0	146	1	73	25.0%	0.05 [0.00, 3.18]
NCT00665925	0	304	0	153		Not estimable
NCT00831922	0	22	0	18		Not estimable
NCT01197521	0	614	1	304	24.9%	0.05 [0.00, 3.14]
NCT01197534	0	606	0	302		Not estimable
NCT01197755	1	213	1	109	50.2%	0.49 [0.03, 9.11]
NCT01264770	0	159	0	52		Not estimable
NCT01563978	0	68	0	67		Not estimable
NCT01569074	0	130	0	33		Not estimable
NCT02092961	0	33	0	29		Not estimable
**Total (95% CI)**		**2437**		**1187**	**100.0%**	**0.16 [0.02, 1.24]**
Total events	1		3			

Heterogeneity: $Chi^2 = 1.16$, $df = 2$ ($P = 0.56$); $I^2 = 0\%$
Test for overall effect: $Z = 1.76$ ($P = 0.08$)

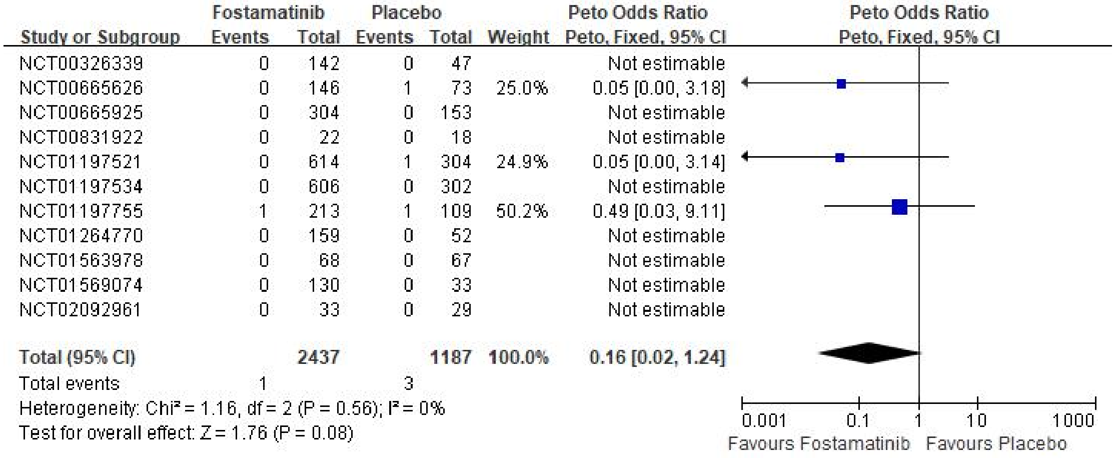

sFig.2 Forest plot of all-cause death based on fostamatinib versus placebo.

Study or Subgroup	Fostamatinib Events	Fostamatinib Total	Placebo Events	Placebo Total	Weight	Peto Odds Ratio Peto, Fixed, 95% CI
NCT00326339	0	142	0	47		Not estimable
NCT00665626	1	146	0	73	14.3%	4.48 [0.07, 286.49]
NCT00665925	0	304	1	153	14.3%	0.05 [0.00, 3.21]
NCT01197521	2	614	0	304	28.5%	4.47 [0.23, 85.04]
NCT01197534	1	606	1	302	28.5%	0.47 [0.02, 8.92]
NCT01197755	1	213	0	109	14.4%	4.53 [0.07, 285.33]
NCT01264770	0	159	0	52		Not estimable
NCT01563978	0	68	0	67		Not estimable
NCT01569074	0	130	0	33		Not estimable
NCT02092961	0	33	0	29		Not estimable
**Total (95% CI)**		**2415**		**1169**	**100.0%**	**1.24 [0.26, 5.97]**
Total events	5		2			

Heterogeneity: Chi² = 4.17, df = 4 (P = 0.38); I² = 4%
Test for overall effect: Z = 0.27 (P = 0.79)

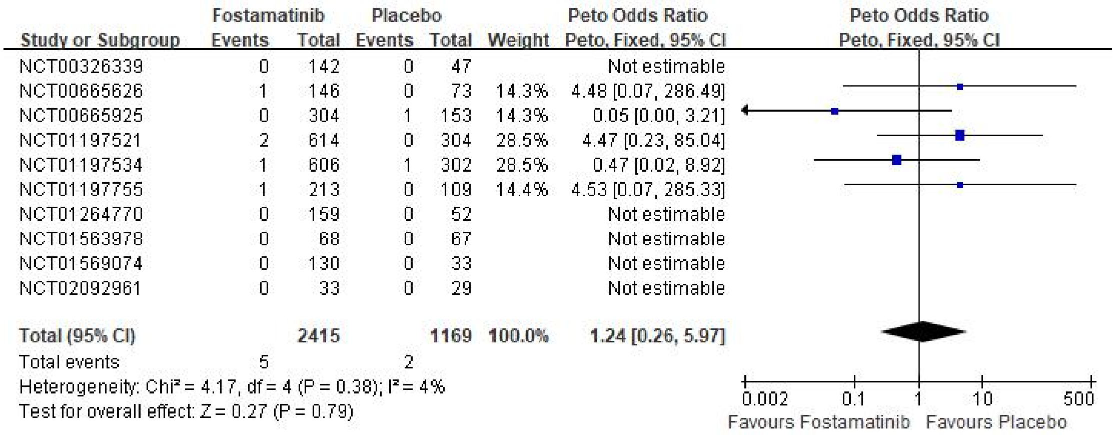

sFig.3 Forest plot of MACE based on fostamatinib versus placebo.

Study or Subgroup	Fostamatinib 100mg bid Events	Fostamatinib 100mg bid Total	Fostamatinib 150mg qd Events	Fostamatinib 150mg qd Total	Weight	Peto Odds Ratio Peto, Fixed, 95% CI
NCT00326339	0	49	0	47		Not estimable
NCT00665925	0	152	0	152		Not estimable
NCT00831922	0	22	0	18		Not estimable
NCT01197521	0	614	2	304	24.6%	0.05 [0.00, 0.93]
NCT01197534	2	610	0	298	24.5%	4.44 [0.23, 85.06]
NCT01197755	0	105	1	108	13.9%	0.14 [0.00, 7.02]
NCT01242514	3	1343	1	357	36.9%	0.79 [0.07, 8.73]
NCT01264770	0	81	0	73		Not estimable
NCT01569074	0	31	0	33		Not estimable
**Total (95% CI)**		**3007**		**1390**	**100.0%**	**0.48 [0.11, 2.05]**
Total events	5		4			

Heterogeneity: Chi² = 5.04, df = 3 (P = 0.17); I² = 40%
Test for overall effect: Z = 1.00 (P = 0.32)

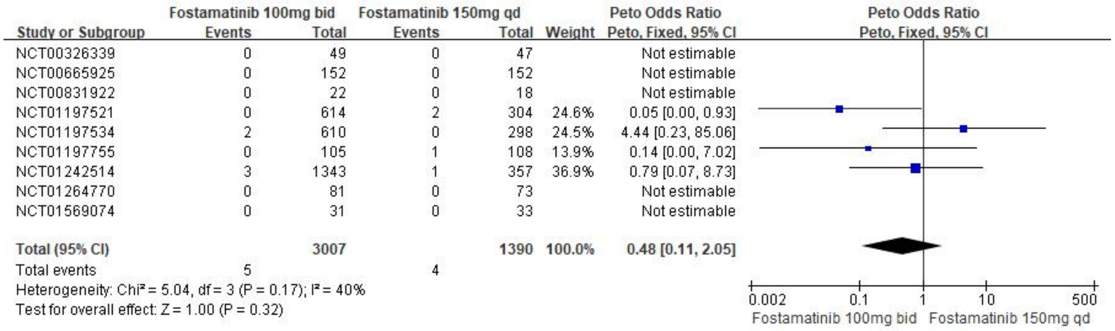

sFig.4 Forest plot of MACE based on fostamatinib 100mg bid versus fostamatinib 150mg qd.

Study or Subgroup	Fostamatinib Events	Fostamatinib Total	Placebo Events	Placebo Total	Weight	Peto Odds Ratio Peto, Fixed, 95% CI
NCT00326339	0	142	0	47		Not estimable
NCT00665626	0	146	0	73		Not estimable
NCT00665925	1	304	1	153	26.1%	0.48 [0.03, 8.99]
NCT01197521	1	614	0	304	13.0%	4.46 [0.07, 287.06]
NCT01197534	1	606	0	302	13.0%	4.47 [0.07, 286.68]
NCT01197755	1	213	1	109	26.2%	0.49 [0.03, 9.11]
NCT01264770	2	159	0	52	21.7%	3.79 [0.15, 95.30]
NCT01563978	0	68	0	67		Not estimable
NCT01569074	0	130	0	33		Not estimable
NCT02092961	0	33	0	29		Not estimable
**Total (95% CI)**		**2415**		**1169**	**100.0%**	**1.34 [0.30, 6.03]**
Total events	6		2			

Heterogeneity: Chi² = 1.98, df = 4 (P = 0.74); I² = 0%
Test for overall effect: Z = 0.39 (P = 0.70)

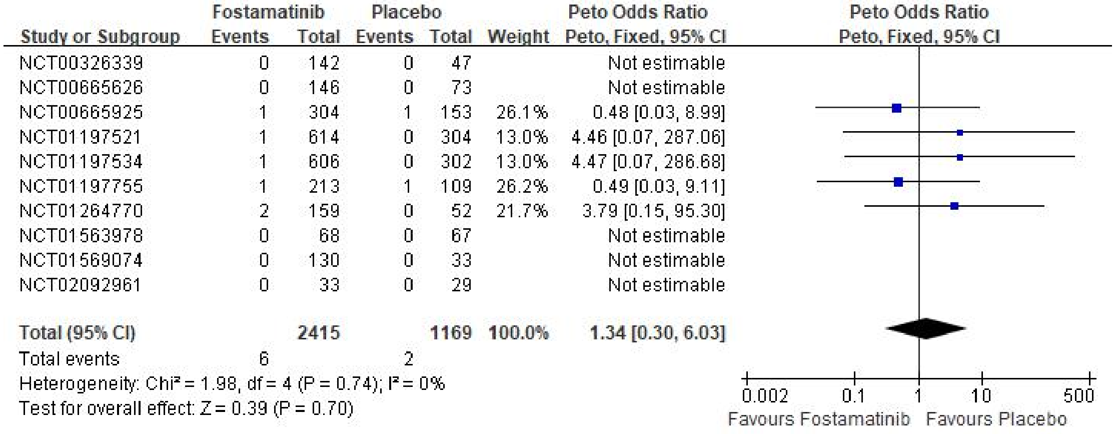

sFig.5 Forest plot of ischaemic heart disease based on fostamatinib versus placebo.

Study or Subgroup	Fostamatinib 100mg bid Events	Fostamatinib 100mg bid Total	Fostamatinib 150mg qd Events	Fostamatinib 150mg qd Total	Weight	Peto Odds Ratio Peto, Fixed, 95% CI
NCT00326339	0	49	0	47		Not estimable
NCT00665925	0	152	1	152	11.2%	0.14 [0.00, 6.82]
NCT00831922	0	22	0	18		Not estimable
NCT01197521	0	614	1	304	9.9%	0.05 [0.00, 3.14]
NCT01197534	2	610	0	298	19.7%	4.44 [0.23, 85.06]
NCT01197755	0	105	1	108	11.2%	0.14 [0.00, 7.02]
NCT01242514	4	1343	1	357	37.0%	1.06 [0.12, 9.16]
NCT01264770	1	81	0	73	11.1%	6.69 [0.13, 339.16]
NCT01569074	0	31	0	33		Not estimable
**Total (95% CI)**		**3007**		**1390**	**100.0%**	**0.81 [0.22, 2.99]**
Total events	7		4			

Heterogeneity: Chi² = 5.77, df = 5 (P = 0.33); I² = 13%
Test for overall effect: Z = 0.32 (P = 0.75)

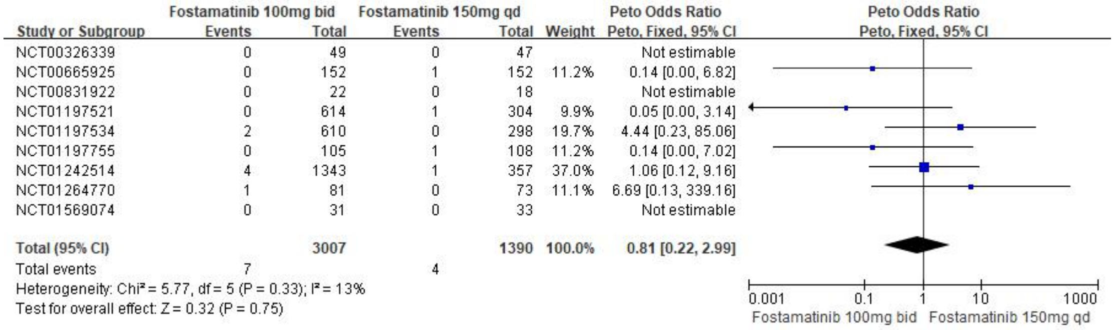

sFig.6 Forest plot of ischaemic heart disease based on fostamatinib 100mg bid versus fostamatinib 150mg qd.

Study or Subgroup	Fostamatinib Events	Fostamatinib Total	Placebo Events	Placebo Total	Weight	Peto Odds Ratio Peto, Fixed, 95% CI
NCT00326339	0	142	0	47		Not estimable
NCT00665626	1	146	0	73	33.3%	4.48 [0.07, 286.49]
NCT00665925	0	304	1	153	33.4%	0.05 [0.00, 3.21]
NCT01197521	1	614	0	304	33.2%	4.46 [0.07, 287.06]
NCT01197534	0	606	0	302		Not estimable
NCT01197755	0	213	0	109		Not estimable
NCT01264770	0	159	0	52		Not estimable
NCT01563978	0	68	0	67		Not estimable
NCT01569074	0	130	0	33		Not estimable
NCT02092961	0	33	0	29		Not estimable
**Total (95% CI)**		**2415**		**1169**	**100.0%**	**1.00 [0.09, 11.02]**
Total events	2		1			

Heterogeneity: Chi² = 2.98, df = 2 (P = 0.23); I² = 33%
Test for overall effect: Z = 0.00 (P = 1.00)

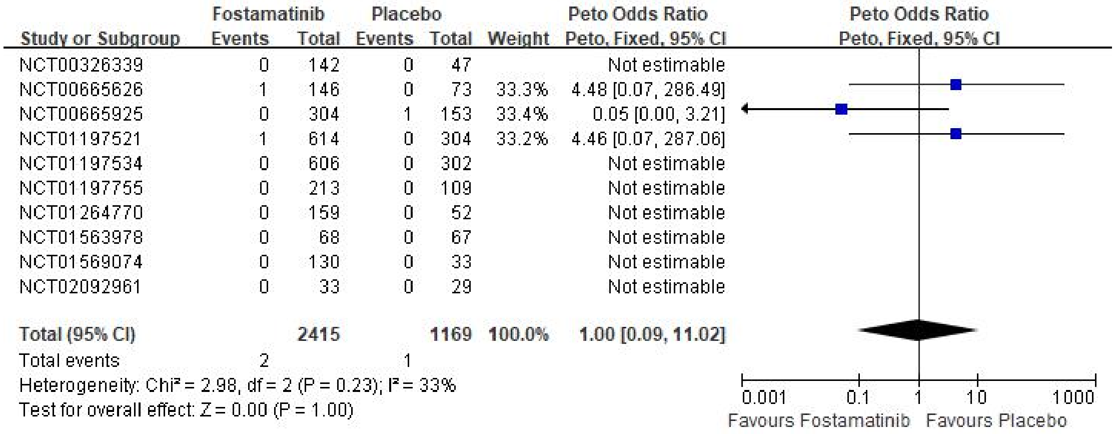

sFig.7 Forest plot of stroke based on fostamatinib versus placebo.

Study or Subgroup	Fostamatinib 100mg bid Events	Fostamatinib 100mg bid Total	Fostamatinib 150mg qd Events	Fostamatinib 150mg qd Total	Weight	Peto Odds Ratio Peto, Fixed, 95% CI
NCT00326339	0	49	0	47		Not estimable
NCT00665925	0	152	0	152		Not estimable
NCT00831922	0	22	0	18		Not estimable
NCT01197521	0	614	1	304	57.2%	0.05 [0.00, 3.14]
NCT01197534	0	610	0	298		Not estimable
NCT01197755	0	105	0	108		Not estimable
NCT01242514	1	1343	0	357	42.8%	3.55 [0.03, 436.08]
NCT01264770	0	81	0	73		Not estimable
NCT01569074	0	31	0	33		Not estimable
**Total (95% CI)**		**3007**		**1390**	**100.0%**	**0.31 [0.01, 7.13]**
Total events	1		1			

Heterogeneity: Chi² = 1.74, df = 1 (P = 0.19); I² = 43%
Test for overall effect: Z = 0.74 (P = 0.46)

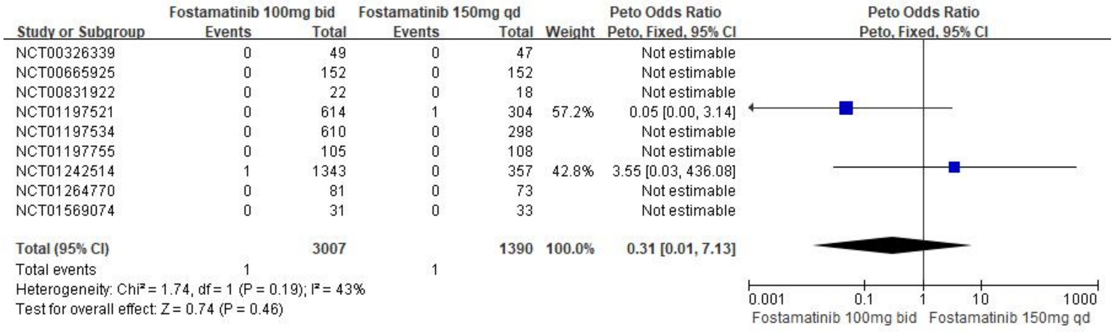

sFig.8 Forest plot of stroke based on fostamatinib 100mg bid versus fostamatinib 150mg qd.

	Fostamatinib		Placebo			Odds Ratio
Study or Subgroup	Events	Total	Events	Total	Weight	M-H, Fixed, 95% CI
NCT00326339	0	142	0	47		Not estimable
NCT00665626	0	146	0	73		Not estimable
NCT00665925	0	304	0	153		Not estimable
NCT01197521	1	614	0	304	47.3%	1.49 [0.06, 36.66]
NCT01197534	0	606	0	302		Not estimable
NCT01197755	0	213	0	109		Not estimable
NCT01264770	1	159	0	52	52.7%	0.99 [0.04, 24.77]
NCT01563978	0	68	0	67		Not estimable
NCT01569074	0	130	0	33		Not estimable
NCT02092961	0	33	0	29		Not estimable
**Total (95% CI)**		**2415**		**1169**	**100.0%**	**1.23 [0.13, 11.87]**
Total events	2		0			

Heterogeneity: $Chi^2 = 0.03$, df = 1 (P = 0.86); $I^2 = 0\%$
Test for overall effect: Z = 0.18 (P = 0.86)

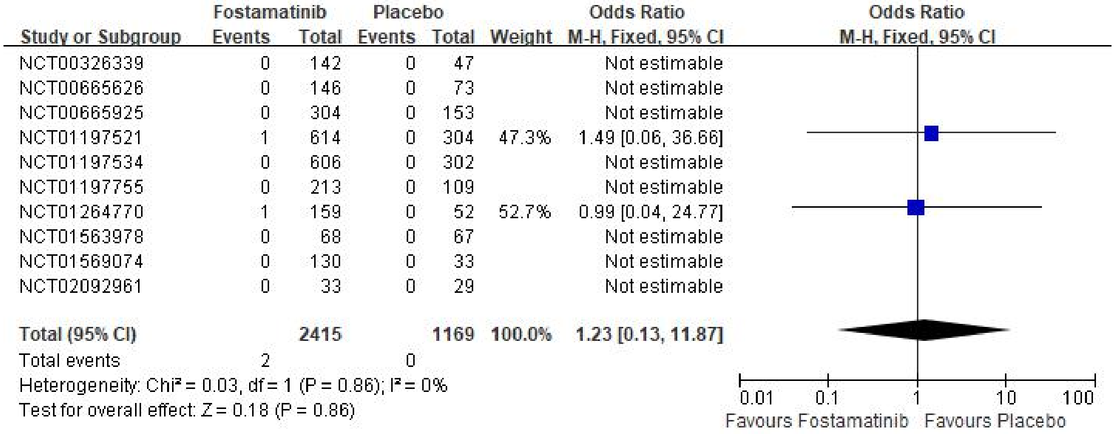

sFig.9 Forest plot of pulmonary heart disease and disease of pulmonary circulation based on fostamatinib versus placebo.

	Fostamatinib 100mg bid		Fostamatinib 150mg qd			Peto Odds Ratio
Study or Subgroup	Events	Total	Events	Total	Weight	Peto, Fixed, 95% CI
NCT00326339	0	49	0	47		Not estimable
NCT00665925	0	152	0	152		Not estimable
NCT00831922	0	22	0	18		Not estimable
NCT01197521	0	614	1	304	57.2%	0.05 [0.00, 3.14]
NCT01197534	0	610	0	298		Not estimable
NCT01197755	0	105	0	108		Not estimable
NCT01242514	1	1343	0	357	42.8%	3.55 [0.03, 436.08]
NCT01264770	0	81	0	73		Not estimable
NCT01569074	0	31	0	33		Not estimable
**Total (95% CI)**		**3007**		**1390**	**100.0%**	**0.31 [0.01, 7.13]**
Total events	1		1			

Heterogeneity: $Chi^2 = 1.74$, df = 1 (P = 0.19); $I^2 = 43\%$
Test for overall effect: Z = 0.74 (P = 0.46)

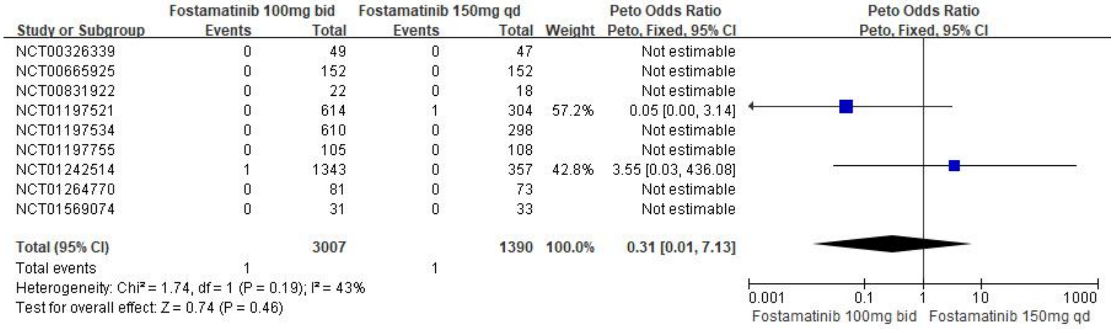

sFig.10 Forest plot of pulmonary heart disease and disease of pulmonary circulation based on fostamatinib 100mg bid versus fostamatinib 150mg qd.

Study or Subgroup	Fostamatinib Events	Fostamatinib Total	Placebo Events	Placebo Total	Weight	Peto Odds Ratio Peto, Fixed, 95% CI
NCT00326339	0	142	0	47		Not estimable
NCT00665626	0	146	0	73		Not estimable
NCT00665925	0	304	0	153		Not estimable
NCT01197521	5	614	1	304	35.0%	2.11 [0.38, 11.61]
NCT01197534	7	606	0	302	40.8%	4.52 [0.93, 21.89]
NCT01197755	1	213	0	109	5.9%	4.53 [0.07, 285.33]
NCT01264770	2	159	0	52	9.8%	3.79 [0.15, 95.30]
NCT01563978	0	68	0	67		Not estimable
NCT01569074	0	130	2	33	8.5%	0.01 [0.00, 0.22]
NCT02092961	0	33	0	29		Not estimable
**Total (95% CI)**		**2415**		**1169**	**100.0%**	**1.96 [0.72, 5.38]**
Total events	15		3			

Heterogeneity: Chi² = 11.62, df = 4 (P = 0.02); I² = 66%
Test for overall effect: Z = 1.31 (P = 0.19)

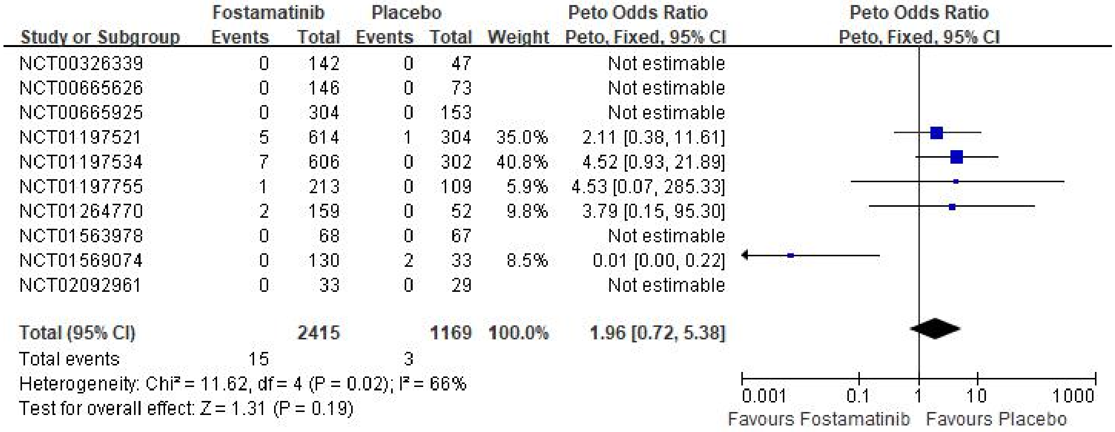

sFig.11 Forest plot of other forms of heart disease based on fostamatinib versus placebo.

Study or Subgroup	Fostamatinib 100mg bid Events	Fostamatinib 100mg bid Total	Fostamatinib 150mg qd Events	Fostamatinib 150mg qd Total	Weight	Peto Odds Ratio Peto, Fixed, 95% CI
NCT00326339	0	49	0	47		Not estimable
NCT00665925	0	152	0	152		Not estimable
NCT00831922	0	22	0	18		Not estimable
NCT01197521	1	614	5	304	30.6%	0.10 [0.02, 0.56]
NCT01197534	6	610	2	298	40.6%	1.43 [0.32, 6.29]
NCT01197755	0	105	1	108	5.8%	0.14 [0.00, 7.02]
NCT01242514	6	1343	0	357	23.0%	3.56 [0.50, 25.46]
NCT01264770	0	81	0	73		Not estimable
NCT01569074	0	31	0	33		Not estimable
**Total (95% CI)**		**3007**		**1390**	**100.0%**	**0.69 [0.27, 1.76]**
Total events	13		8			

Heterogeneity: Chi² = 9.06, df = 3 (P = 0.03); I² = 67%
Test for overall effect: Z = 0.78 (P = 0.44)

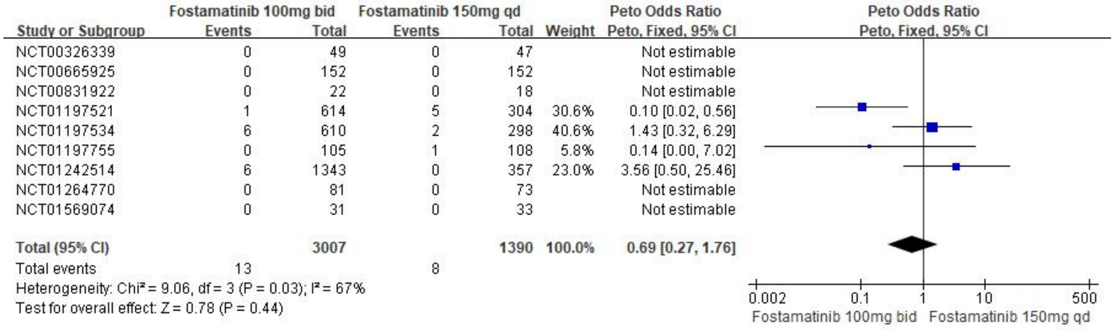

sFig.12 Forest plot of other forms of heart disease based on fostamatinib 100mg bid versus fostamatinib 150mg qd.

Study or Subgroup	Fostamatinib Events	Fostamatinib Total	Placebo Events	Placebo Total	Weight	Risk Ratio M-H, Fixed, 95% CI
**1.1.1 4 weeks**						
NCT01563978	3	68	1	67	1.5%	2.96 [0.32, 27.71]
**Subtotal (95% CI)**		**68**		**67**	**1.5%**	**2.96 [0.32, 27.71]**
Total events	3		1			
Heterogeneity: Not applicable						
Test for overall effect: Z = 0.95 (P = 0.34)						
**1.1.2 12 weeks**						
NCT00326339	5	142	0	47	1.1%	3.69 [0.21, 65.55]
NCT00665626	19	146	3	73	5.8%	3.17 [0.97, 10.36]
NCT01569074	23	130	2	33	4.6%	2.92 [0.72, 11.76]
**Subtotal (95% CI)**		**418**		**153**	**11.4%**	**3.12 [1.32, 7.38]**
Total events	47		5			
Heterogeneity: Chi² = 0.02, df = 2 (P = 0.99); I² = 0%						
Test for overall effect: Z = 2.59 (P = 0.010)						
**1.1.3 24 weeks**						
NCT00665925	39	304	7	153	13.4%	2.80 [1.28, 6.12]
NCT01197755	29	213	9	109	17.2%	1.65 [0.81, 3.36]
NCT01264770	15	159	0	52	1.1%	10.27 [0.63, 168.69]
NCT02092961	4	33	1	29	1.5%	3.52 [0.42, 29.69]
**Subtotal (95% CI)**		**709**		**343**	**33.2%**	**2.48 [1.51, 4.10]**
Total events	87		17			
Heterogeneity: Chi² = 2.46, df = 3 (P = 0.48); I² = 0%						
Test for overall effect: Z = 3.56 (P = 0.0004)						
**1.1.4 52 weeks**						
NCT01197521	148	614	12	304	23.1%	6.11 [3.45, 10.82]
NCT01197534	123	606	16	302	30.8%	3.83 [2.32, 6.33]
**Subtotal (95% CI)**		**1220**		**606**	**53.9%**	**4.81 [3.30, 7.01]**
Total events	271		28			
Heterogeneity: Chi² = 1.46, df = 1 (P = 0.23); I² = 31%						
Test for overall effect: Z = 8.17 (P < 0.00001)						
**Total (95% CI)**		**2415**		**1169**	**100.0%**	**3.82 [2.88, 5.05]**
Total events	408		51			
Heterogeneity: Chi² = 9.31, df = 9 (P = 0.41); I² = 3%						
Test for overall effect: Z = 9.35 (P < 0.00001)						
Test for subgroup differences: Chi² = 4.48, df = 3 (P = 0.21), I² = 33.1%						

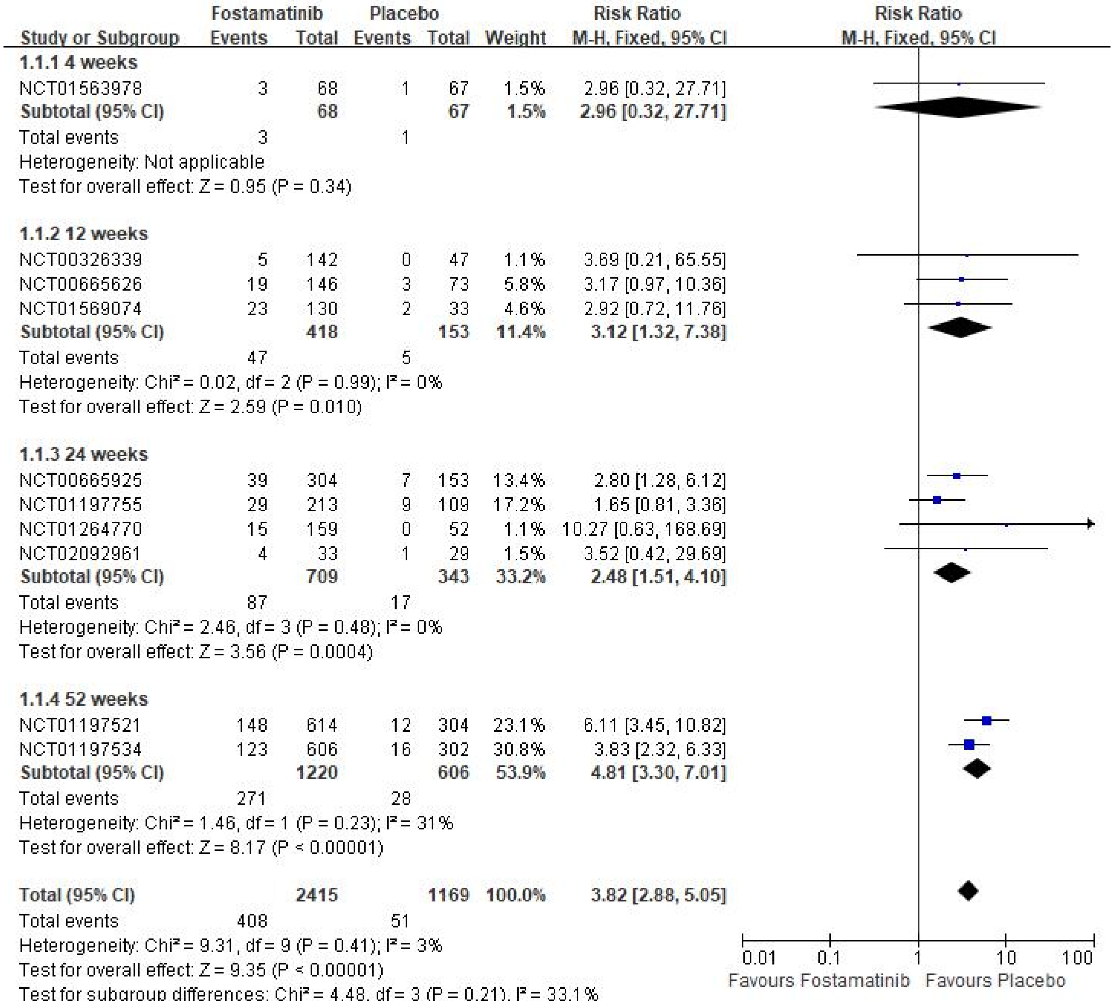

sFig.13 Forest plot of hypertension based on fostamatinib versus placebo.

Study or Subgroup	Fostamatinib 100mg bid Events	Fostamatinib 100mg bid Total	Fostamatinib 150mg qd Events	Fostamatinib 150mg qd Total	Weight	Risk Ratio M-H, Fixed, 95% CI
**1.3.1 12 weeks**						
NCT00326339	3	95	2	47	1.0%	0.74 [0.13, 4.29]
NCT01569074	17	97	6	33	3.4%	0.96 [0.41, 2.24]
**Subtotal (95% CI)**		**192**		**80**	**4.4%**	**0.91 [0.43, 1.95]**
Total events	20		8			
Heterogeneity: Chi² = 0.07, df = 1 (P = 0.79); I² = 0%						
Test for overall effect: Z = 0.24 (P = 0.81)						
**1.3.2 24 weeks**						
NCT00665925	21	152	18	152	6.7%	1.17 [0.65, 2.10]
NCT01197755	14	105	15	108	5.5%	0.96 [0.49, 1.89]
NCT01264770	11	81	10	73	3.9%	0.99 [0.45, 2.20]
**Subtotal (95% CI)**		**338**		**333**	**16.2%**	**1.05 [0.72, 1.55]**
Total events	46		43			
Heterogeneity: Chi² = 0.21, df = 2 (P = 0.90); I² = 0%						
Test for overall effect: Z = 0.26 (P = 0.79)						
**1.3.3 52 weeks**						
NCT01197521	85	614	76	304	38.1%	0.55 [0.42, 0.73]
NCT01197534	85	610	55	298	27.7%	0.75 [0.55, 1.03]
**Subtotal (95% CI)**		**1224**		**602**	**65.8%**	**0.64 [0.52, 0.78]**
Total events	170		131			
Heterogeneity: Chi² = 2.14, df = 1 (P = 0.14); I² = 53%						
Test for overall effect: Z = 4.27 (P < 0.0001)						
**1.3.4 109 weeks**						
NCT01242514	197	1343	23	357	13.6%	2.28 [1.50, 3.45]
**Subtotal (95% CI)**		**1343**		**357**	**13.6%**	**2.28 [1.50, 3.45]**
Total events	197		23			
Heterogeneity: Not applicable						
Test for overall effect: Z = 3.88 (P = 0.0001)						
**Total (95% CI)**		**3097**		**1372**	**100.0%**	**0.94 [0.80, 1.10]**
Total events	433		205			
Heterogeneity: Chi² = 33.95, df = 7 (P < 0.0001); I² = 79%						
Test for overall effect: Z = 0.74 (P = 0.46)						
Test for subgroup differences: Chi² = 30.17, df = 3 (P < 0.00001), I² = 90.1%						

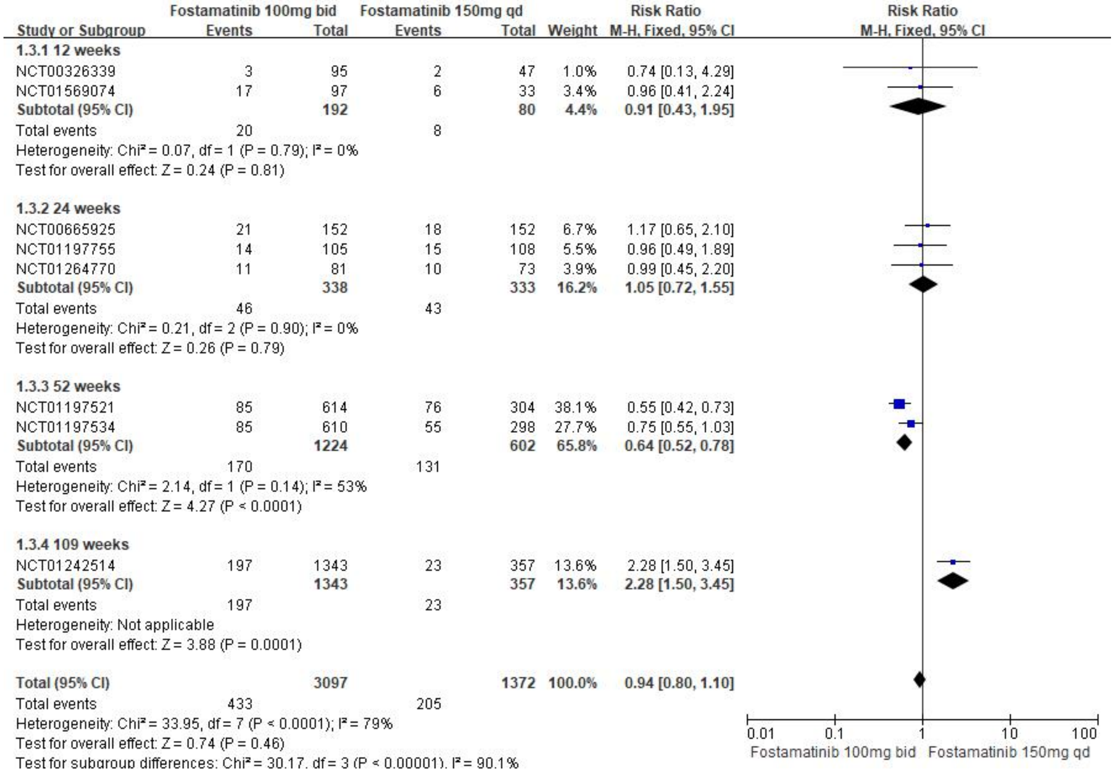

sFig.14 Forest plot of hypertension based on fostamatinib 100mg bid versus fostamatinib 150mg qd.

Study or Subgroup	Fostamatinib 100mg bid Events	Fostamatinib 100mg bid Total	Fostamatinib 100mg qd Events	Fostamatinib 100mg qd Total	Weight	Risk Ratio M-H, Fixed, 95% CI
NCT01242514	197	1343	15	212	81.5%	2.07 [1.25, 3.43]
NCT01264770	11	81	5	57	18.5%	1.55 [0.57, 4.21]
**Total (95% CI)**		**1424**		**269**	**100.0%**	**1.98 [1.26, 3.11]**
Total events	208		20			
Heterogeneity: Chi² = 0.26, df = 1 (P = 0.61); I² = 0%						
Test for overall effect: Z = 2.95 (P = 0.003)						

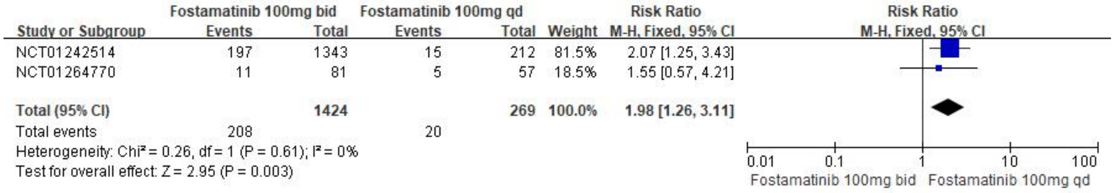

sFig.15 Forest plot of hypertension based on fostamatinib 100mg bid versus fostamatinib 100mg qd.

Study or Subgroup	Fostamatinib Events	Fostamatinib Total	Adamumab Events	Adamumab Total	Weight	Risk Ratio M-H, Fixed, 95% CI
NCT01264770	26	211	5	54	92.4%	1.33 [0.54, 3.30]
NCT02092961	5	55	0	28	7.6%	5.70 [0.33, 99.48]
**Total (95% CI)**		**266**		**82**	**100.0%**	**1.66 [0.71, 3.91]**
Total events	31		5			
Heterogeneity: Chi² = 0.94, df = 1 (P = 0.33); I² = 0%						
Test for overall effect: Z = 1.17 (P = 0.24)						

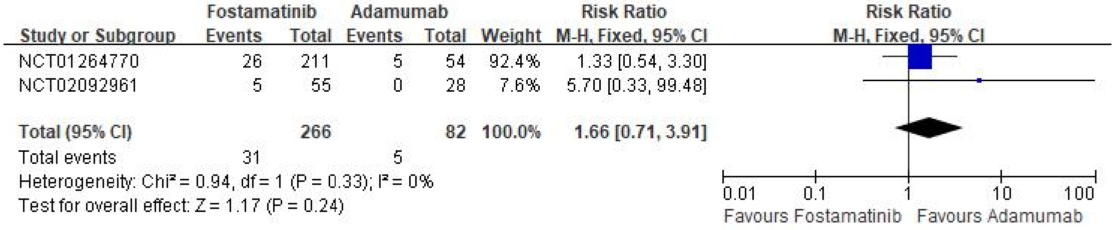

sFig.16 Forest plot of hypertension based on fostamatinib versus adamumab.
